# Microstates and connectivity states reveal task-related network reorganization in Alzheimer's disease patients

**DOI:** 10.1016/j.cnp.2026.07.016

**Published:** 2026-07-28

**Authors:** Ji-Won Kim, Azamat Yeldesbay, Christina Kehm, Ronja V. Fassbender, Oezguer A. Onur, Nils Rosjat, Gereon R. Fink, Silvia Daun

**Affiliations:** a Cognitive Neuroscience, Institute of Neuroscience & Medicine (INM-3), Forschungszentrum Jülich; b Department of Neurology, Faculty of Medicine, University Hospital Cologne, Germany; c Institute of Zoology, University of Cologne, Germany

**Keywords:** RS-EEG, Functional connectivity, Dementia, TMS, Phase-locking value

## Abstract

**Objective:**

Microstates derived from EEG amplitude and connectivity states from EEG phase examine distinct aspects of the brain's dynamic organization. However, it is unclear how sensitive they are to changes in functional connectivity in Alzheimer's disease (AD). We examined pre- and post-task alterations of brain activity in AD patients to compare the ability of microstates and connectivity states to capture disease-related network dysfunction.

**Methods:**

Resting-state EEG (RS-EEG) was recorded before and after a memory task in fifteen patients with AD and fifteen healthy controls. During the memory task, either low-intensity repetitive transcranial magnetic stimulation (rTMS) over the parieto-occipital region or sham stimulation was applied. We quantified microstates and phase-based connectivity states in the alpha frequency range from the RS-EEG.

**Results:**

While microstates showed task-related alterations in healthy controls only, connectivity states were more sensitive to alterations in the AD group. rTMS appeared to reduce these task-related alterations in connectivity states. Connectivity states indicated a shift towards lower, more diffuse connectivity in AD patients. Connectivity states were correlated with memory task performance and amyloid-beta levels in the AD group.

**Conclusions:**

Connectivity states indicated decreased task-related network stability in AD patients, which correlated with memory task performance. The findings support the neural efficiency hypothesis, which states that more efficient brain network optimization during tasks is linked to better performance.

**Significance:**

Given the progressive decline of alpha power in AD patients, phase-based connectivity states provide valuable complementary information to microstates and may serve as a biomarker for assessing large-scale network alterations in relation to disease progression.

## Highlights

• EEG connectivity states (CS) as a novel method to detect large-scale network changes.
• Decrease in connectivity and task-related network stability in Alzheimer‘s patients.
• Microstates and CS are complementary biomarkers of disease progression.

## 1 Introduction

Alzheimer's disease (AD) is a neurodegenerative disorder characterized by the progressive decline of cognitive functions. Disease progression in AD is accompanied by alterations in functional network organization (Ranasinghe and Mapa, 2024; Sheline and Raichle, 2013). Among the various functional connectivity measures, dynamic connectivity states have emerged as a promising tool for the analysis of large-scale network patterns in resting-state (RS) data (Calhoun et al., 2014; Rosjat et al., 2024). Connectivity states are attained by computing functional connectivity maps in a sliding window approach along the time scale and identifying the main topographical features among them. In AD patients with dementia, fMRI-derived connectivity states showed alterations compared to healthy controls and patients with mild cognitive impairment (MCI) (Schumacher et al., 2019a; Zang et al., 2024). A study using a machine learning based classification algorithm revealed alterations in fMRI connectivity states in subjects with MCI that corresponded to cognitive decline, indicating the possibility to utilize connectivity states as biomarkers for identifying MCI subjects at high risk of converting to dementia (Jing et al., 2023). Despite these intriguing results from fMRI connectivity states, to date, studies examining EEG connectivity states in AD patients are lacking. Although a one-to-one comparison between EEG- and fMRI-based connectivity states is not meaningful, as the two modalities reflect distinct physiological processes and time scales, EEG may constitute a valuable alternative to fMRI for assessing aberrant connectivity states due to its cost-effectiveness and ubiquitous availability.

Regarding EEG analyses, various studies investigated microstates and explored their potential in demonstrating network configurations in AD patients (Schumacher et al., 2019b; Smailovic et al., 2019; Tait et al., 2020). Similar to connectivity states, microstates represent time-varying topographical patterns, but are computed from EEG amplitude values (Lehmann et al., 1987; Michel and Koenig, 2018). While the optimal number of microstates needs to be determined for each specific dataset, many studies showed consistent topographies for the first four, which are often referred to as the four “canonical” microstates A, B, C, and D (Koenig et al., 2024). Studies in patients with AD and MCI revealed a tendency for increased duration and coverage of the asymmetrical microstates A and B and decreased duration and coverage of the symmetrical microstate C (Lassi et al., 2023; Lian et al., 2021; Musaeus et al., 2019; Smailovic et al., 2019). Notably, this pattern differed from what had previously been described in healthy aging (Koenig et al., 2002). Studies comparing microstates during RS-EEG before and after a task demonstrated task-related differences (D'Croz-Baron et al., 2021; Murphy et al., 2018), suggesting that microstates reflect both long-term (i.e., disease-related) changes and short-term (i.e., task-related) alterations of network topography.

However, since the computation of microstates is amplitude-based, any information regarding phase-based connectivity is lost. In a recent study examining EEG phase-based connectivity states in comparison to microstates in healthy subjects, connectivity states were shown to be reproducible over multiple recording sessions, similar to microstates (Rosjat et al., 2024). Notably, connectivity states were more sensitive to motor task-related differences than microstates and revealed a post-task suppression of frontal activity. The divergence between amplitude- and phase-based connectivity has been ascribed to local synchronization within a neural population driving the EEG amplitude, while EEG phase reflects the synchronization of oscillatory activity between different neural populations (Daffertshofer and van Wijk, 2011; Siems and Siegel, 2020).

Transcranial magnetic stimulation (TMS) has been extensively investigated for neuromodulation and -enhancement (Alfihed et al., 2024). Several studies reported beneficial repetitive TMS (rTMS) effects on memory and mood (Abualait et al., 2021; Bashir et al., 2022; Di Lazzaro et al., 2021), attributed to the influence of TMS on cortical excitability and trans-regional connectivity (Di Lazzaro et al., 2021). Microstates were able to capture TMS-related effects in healthy subjects and AD patients (Croce et al., 2018; Hanoglu et al., 2022). However, we could not find a study examining the effect of rTMS on connectivity states in patients with Alzheimer's clinical syndrome.

We here studied microstates and connectivity states in the alpha frequency from RS-EEG in AD patients and age-matched healthy controls (HC). We evaluated task-related differences between RS sessions before and after a memory task. The memory task was conducted with concurrent rTMS adjacent to the upper alpha frequency over the bilateral parieto-occipital brain areas or sham stimulation. We determined whether connectivity states from EEG data resemble those obtained from fMRI data, which showed a shift towards lower and more diffuse connectivity in AD patients (Kuzu et al., 2026; Schumacher et al., 2019a). We further aimed to compare the ability of microstates and connectivity states to detect task-related alterations in network dynamics. As the alpha amplitude decreases with disease progression in AD (Lejko et al., 2020), we hypothesized phase-based connectivity states to be more sensitive to task-related alterations than amplitude-based microstates. We further hypothesized to detect rTMS-induced increases in microstates and connectivity states reflecting alpha synchronization in the posterior regions through TMS. The dataset used was originally collected for another study examining the association between rTMS and memory performance (Fassbender et al., 2026). To avoid overlapping reporting, specific aspects of the data were not analyzed.

## 2 Materials & methods

### 2.1 Subjects

Fifteen subjects (aged 53 to 84 years) for the AD group and 15 healthy, age-matched controls (HC) were recruited through the University Hospital of Cologne (Table 1). Subjects were included in the AD group if they featured an AT(N) biomarker profile associated with the AD continuum as defined by the diagnostic guidelines proposed by the NIA-AA (Jack et al., 2018). Exclusion criteria were the presence of psychiatric, neuroinflammatory, or neurodegenerative diseases other than AD, and epilepsy. All subjects gave written informed consent. The study was approved by the Ethics Committee of the University of Cologne (No. 18–336) and conducted following the Declaration of Helsinki.

**Table 1:** Subject groups.

	AD (*N* = 15)	HC (N = 15)
Age, mean ± SD	72.73 ± 8.28	72.93 ± 7.17
Sex	5 female, 10 male	7 female, 8 male
Years of education, mean ± SD	14.73 ± 3.06	15.87 ± 3.46
MMSE, mean ± SD	24.93 ± 3.31	28.93 ± 0.96
GDS, mean ± SD	2.27 ± 1.98	0.27 ± 0.46

AD: Alzheimer's disease, GDS: Geriatric depression scale, HC: Healthy controls, MMSE: Mini mental state examination, SD: Standard deviation.

### 2.2 Experimental setup and memory paradigm

**Fig. 1:**
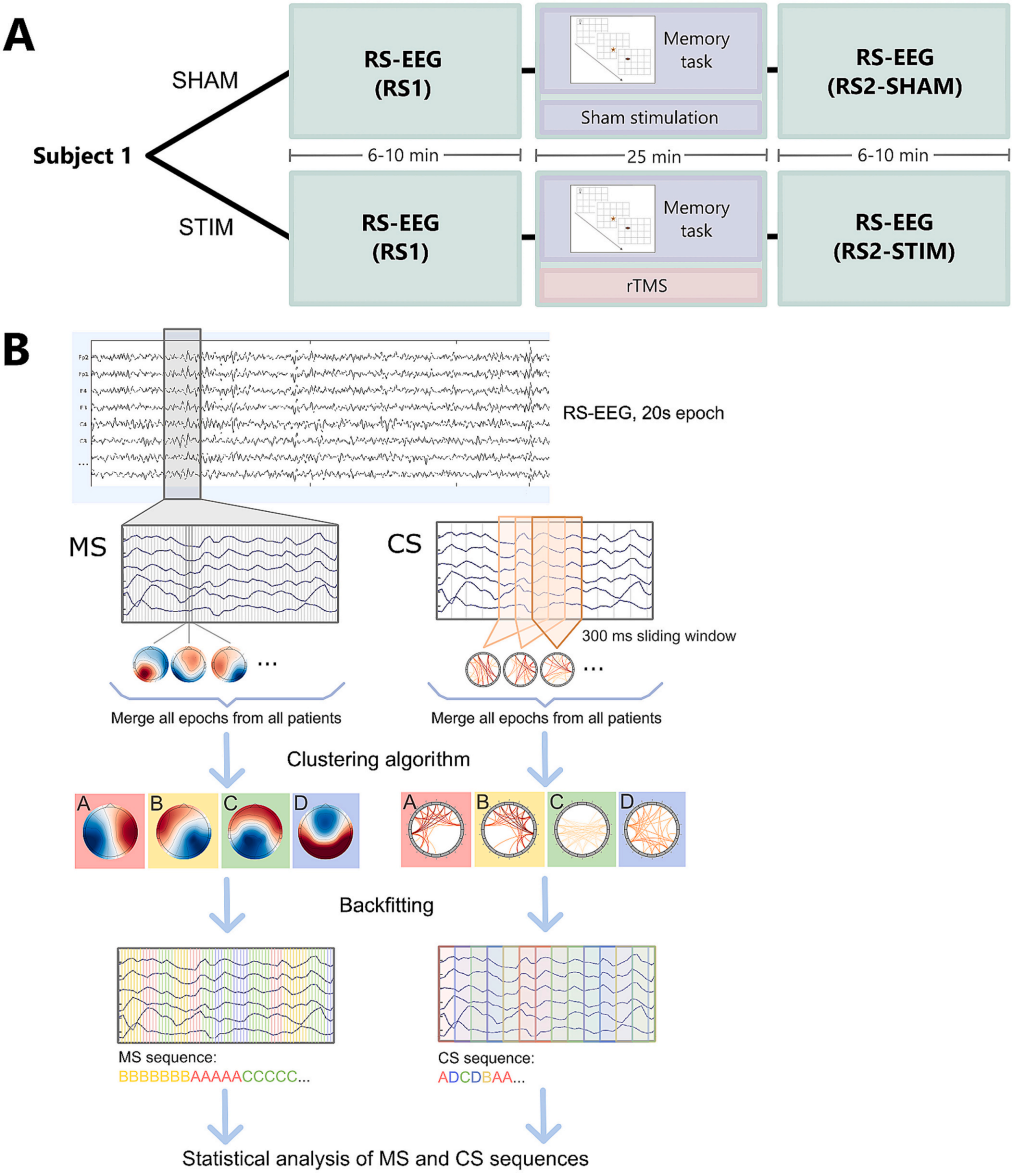
Schematic representation of study sessions and computation of microstates (MS) and connectivity states (CS) A. Each subject underwent two conditions, SHAM and STIM. Both conditions consisted of a RS-EEG (6–10 min) and a memory task (25 min), followed by another RS-EEG (6–10 min). In the SHAM condition, sham stimulation was applied during the memory task. In the STIM condition, rTMS was applied during the memory task. B. Each RS-EEG was divided into 20 s artifact-free epochs. For microstates (MS), a power topography plot was computed for each timepoint. Topography plots from all epochs and subjects were pooled together and entered into a clustering algorithm, which defined four global microstates. The backfitting of each timepoint then resulted in microstate sequences. For connectivity states (CS), ciPLV was computed over sliding windows of 300 ms with an overlap of 200 ms, resulting in connectivity plots with a time resolution of 100 ms. Connectivity plots were pooled together from all epochs and subjects and entered into a clustering algorithm, which defined four global connectivity states. The backfitting of each time window resulted in connectivity state sequences.

All subjects participated in an experimental paradigm consisting of two conditions (STIM and SHAM) with three sessions each (RS1, memory task, RS2) (Fig. 1A). The order of the two conditions was randomized. During RS1 and RS2, continuous RS-EEG was recorded for 6–10 min with the patients' eyes closed. EEG was recorded by 19 active Ag/AgCl electrodes in an elastic cap (MEDCAP) placed according to the 10–20 system and two additional channels for EOG recording. A ground electrode Fpz was placed on the forehead. Cz was defined as the online reference channel. EEG was amplified by the Neurowerk Amplifier (Headbox DB26, Neurowerk EEG software V13.19.2.73). The sampling rate was 512 Hz.

Between RS1 and RS2, subjects participated in a visuospatial memory task. First, subjects were shown ten different images of natural or everyday household objects. Each image was placed in a unique position inside a 4 × 5 grid. The presentation lasted for four seconds and was repeated three times. Subjects were instructed to remember the object and its position. Subsequently, twenty images (ten learned and ten new) were presented in sequential order, together with an empty 4 × 5 grid and a separate box. If the subject evaluated the image to be a previously learned object, they were instructed to place the object in its correct grid position. If the object was evaluated as previously unseen, subjects were asked to put it in the box outside of the grid. For each object, subjects had ten seconds to execute their response. The task performance was evaluated using d-prime and error distance. D-prime was defined as d′ = Z (hit rate) − Z (false alarm rate), where *hit rate* was the percentage of correctly remembered learned objects, *false alarm rate* was the percentage of new objects incorrectly evaluated as previously seen, and *Z* represents the distance from the mean of a standard normal distribution in units of standard deviation. Error distance was calculated from the placement of the object on the grid using the distance to the correct position along the x- and the y-axis measured in pixels, and was defined as $\mathrm{error distance}=\sqrt{\mathrm{distance}{\left(x\right)}^{2}+\mathrm{distance}{\left(y\right)}^{2}}$).

### 2.3 Repetitive transcranial magnetic stimulation (rTMS)

During the memory task, subjects received either 25 min of rTMS or sham stimulation. The rTMS consisted of high-frequency (14 Hz), low-intensity (<10 mT) electric pulses applied using four coils over the bilateral parieto-occipital brain areas (Fassbender et al., 2026). The stimulation protocol was established to target and enhance posterior alpha oscillations in our subjects. While many rTMS studies in patients with AD employ 10–20 Hz stimulation over the dorsolateral prefrontal cortex (Zhang et al., 2025), Fassbender et al. (2026) provided the first investigation into the targeted enhancement of posterior alpha oscillations using rTMS in AD patients. Our study performs a secondary analysis of this dataset to explore rTMS-induced effects on network dynamics in the RS-EEG. A decrease of posterior alpha oscillations in patients with Alzheimer's clinical syndrome has consistently been described (Huang et al., 2000), also in direct correlation with cognitive decline (Jeong, 2004). Previous studies indicate that rTMS delivered close to the individual alpha frequency is optimal for driving the entrainment of endogenous alpha oscillations, and concomitantly modulates cognitive performance (Klimesch et al., 2003; Sauseng et al., 2009; Thut et al., 2011). 14 Hz was chosen as the stimulation frequency to minimize spectral overlap with the alpha band (8–12 Hz) and thereby differentiate between potential stimulation-induced artefacts and endogenous alpha oscillations. The pulses were delivered in 14 Hz trains with a variable inter-train interval (40–56 ms), a varying number of pulses per train (5–20) and a variable inter-stimulus interval (ISI, 1.5–3 ms) within each train. The number of pulses per train and ISI were determined individually for each subject using real-time EEG to identify the values associated with the highest power spectral density in the alpha frequency band at the bilateral parieto-occipital electrodes (O1, O2, P3, P4; CERTIS®, Advanced Neuro Systems - ANS; formerly AB Neurotech) during the stimulation.

The rTMS modality was designed to explore low-intensity stimulation in humans and involved an experimental system not yet commonly used in clinical practice. Low-intensity rTMS with intensities below the action potential threshold has been explored to examine the effects of long-term potentiation, mostly in animals (Moretti and Rodger, 2022). In contrast, human rTMS studies typically employ high-intensity rTMS. However, studies examining changes in metabolites suggest that low- and high-intensity rTMS induce distinct neurobiological processes (Grehl et al., 2015; Heath et al., 2018). Low-intensity rTMS is thought to enhance Hebbian plasticity (Hebb, 1949) through subthreshold neural activity, which increases excitability without triggering excessive activation (Grehl et al., 2015; Moretti and Rodger, 2022). This stimulation procedure thus ensures high tolerability and makes low-intensity rTMS a promising tool for sustained neuromodulation.

### 2.4 EEG data analysis

Preprocessing of the EEG data was conducted using EEGLAB (Delorme and Makeig, 2004) in MATLAB 9.12 (R2022a). EEG data were downsampled to 128 Hz. A 0.5–40 Hz bandpass FIR filter was applied. Independent component analysis was used to remove muscle, eye movement, ECG, and line noise artefacts. The continuous EEG data were segmented into 20s epochs and all epochs containing artefacts were discarded after visual inspection. For microstates, another FIR filter was applied to retain only alpha frequencies (8–12 Hz). Channels were re-referenced to the standard average reference.

Further processing was done using Python 3.12.4. For microstates (Fig. 1B, left column), we utilized the Pycrostates library (Férat et al., 2022). We determined one set of four “global” microstate maps from the pooled epochs of both subject groups. A modified k-means algorithm was applied, which disregards polarity of the EEG topography (Pascual-Marqui et al., 1995). To achieve a more objective labeling of the global microstates, we utilized MS-Template-Explorer (Koenig et al., 2024), which enables the comparison of microstate topographies across studies (see Supplementary Fig. 1). Using the same application, we compared the topographical similarities between microstates computed from each group separately (Supplementary Fig. 2), which resulted in very high topographical similarities (95–99%) between the AD and HC group. We therefore opted to compute global microstate maps across groups, as extracting separate sets of maps per group was shown to inflate the type I error (Murphy et al., 2024). We chose the number of global microstates according to the elbow method (Syakur et al., 2018) applied to the global explained variance curve, which resulted in four maps and a global explained variance of 78.92%. The silhouette score was 0.61, indicating good clustering based on the comparison of intra-cluster distance and inter-cluster separation (Rousseeuw, 1987). As a next step, each timepoint in each epoch was back-fitted to the four global microstate maps. A threshold of 3 timepoints (23.4 ms) was set as the minimum length of a microstate segment to prevent spurious fluctuations.

For connectivity states (Fig. 1B, right column), we followed the approach described in Rosjat et al. (2024). EEG epochs were transformed into time-frequency representations using Morlet wavelets in the alpha frequency range 8–12 and a step size of 0.5 Hz. We determined the functional connectivity between two channels by calculating the corrected imaginary part of the phase-locking value (ciPLV) (Bruña et al., 2018). ciPLV between electrodes m and n was computed for each frequency f within the specified range as follows:

ciPLVm,nf=1TIm∑t=1Texpiϕmtf−ϕntf1−1TRe∑t=1Texpiϕmtf−ϕntf2

where $\phi_{m,n}\left(t,f\right)$ is the phase value at time t for frequency f, and T is the number of time points in the time interval of interest (Lachaux et al., 2000). Following this, the resulting values were averaged across the frequency range to obtain a single alpha-band estimate per time interval. ciPLV was calculated using sliding windows with a time interval of 300 ms and an overlap of 200 ms, resulting in a temporal resolution of 100 ms (Rosjat et al., 2024). The sliding window approach was used to capture time-varying functional connectivity with a temporal resolution comparable to that of microstates (Allen et al., 2014), while the 300 ms window provides sufficient timepoints for the stable estimation of phase-coupling. Only the top 30% strongest ciPLV values were retained to remove spurious connections (Rubinov and Sporns, 2010). This procedure resulted in a connectivity matrix, or graph $G_{\tau }$, for the time window τ. For each epoch, we defined a dynamic graph G as an ordered sequence of connectivity matrices, $G=\left(G_{1},G_{2},\ldots ,G_{N_{w}}\right)$, where $N_{w}$ is the total number of time windows in one epoch, i.e., 194 for the present study. The dynamic graphs were fed into a modified k-means algorithm to determine global connectivity states. We used the elbow method on the Calinski-Harabasz score (Calinski and Harabasz, 1974) curve, which determined the optimal number of clusters to be four. Consistent with our microstate analysis, we also explored group-specific clustering of connectivity states (Supplementary Fig. 3). This comparison revealed strong correlations between all four connectivity state pairs (Pearson's r = 0.77 to 0.96), indicating highly similar connectivity topologies between the AD and HC groups. We therefore opted to compute a single set of global connectivity states across both subject groups. Given the high dimensionality of connectivity states (153 edges in our study), pooling all dynamic graphs and computing one set of global connectivity states allowed for a more stable estimation of cluster centroids. A backfitting of each graph to the global connectivity states was performed.

The microstate and connectivity state segmentations were used to calculate the mean duration and coverage of each state. Duration reflected the mean length in time of each state. Coverage was defined as the percentage of each state relative to the total segmentation.

### 2.5 Statistical analyses

A robust linear mixed-effects model (RLMM) was implemented using the robustlmm package (Koller, 2016) in R 4.5.3 to evaluate the duration and coverage of each microstate and connectivity state. Duration and coverage were treated as dependent variables. These values were calculated at the epoch level, resulting in multiple observations per subject. The number of artifact-free epochs per subject per session ranged from 6 to 20, with a median of 14 epochs. Robust estimation was employed to reduce the influence of outliers. Microstate (A, B, C or D), subject group (AD or HC), session (RS1, RS2-STIM or RS2-SHAM), and their interactions were defined as the fixed effects of interest. Age and sex were inserted as covariates. Subject was defined as a random effect to account for inter-subject variability. To evaluate the main and interaction effects for each microstate, Type III Wald Chi-square tests were performed using the *emmeans* package in R (Lenth and Piaskowski, 2026). If the Wald Chi-square test was significant, pairwise post-hoc testing was conducted with estimated marginal means. Effect size was reported using Cohen's *d*. An identical RLMM was applied to evaluate connectivity states, with the connectivity state (A, B, C or D) substituting for the microstate factor as the fixed effect.

To evaluate the association between memory task performance and microstate/connectivity state duration and coverage, we again implemented an RLMM and used data from the AD group only. Data from the HC group was excluded because their performance scores had too little variability (due to overall high levels of performance). The task performance metric (d′ or error distance) and their interaction with microstates/connectivity states (A, B, C or D) were defined as fixed effects of interest in the RLMM. Covariates and random effects remained the same as above.

We further evaluated the association between cerebrospinal fluid (CSF)-measured Amyloid ⁠β⁠ 1–42 (Aβ_42_) levels in our AD group and their microstate and connectivity state variables using an RLMM. Aβ_42_ values were available for 12 out of 15 patients: For the remaining three patients, CSF diagnostics had been conducted externally and/or exact values were missing from the database. The time interval between CSF collection and the RS-EEG recording in months was included as a covariate in the RLMM.

To account for multiple comparisons across RLMMs, all primary *p*-values obtained from the Wald Chi-square tests were corrected using the Benjamini-Hochberg procedure for the false discovery rate (FDR) (Benjamini and Hochberg, 1995). *P*-values from subsequent post-hoc comparisons were adjusted using the Tukey method as implemented in the *emmeans* package.

## 3 Results

### 3.1 Global microstates and connectivity states

**Fig. 2:**
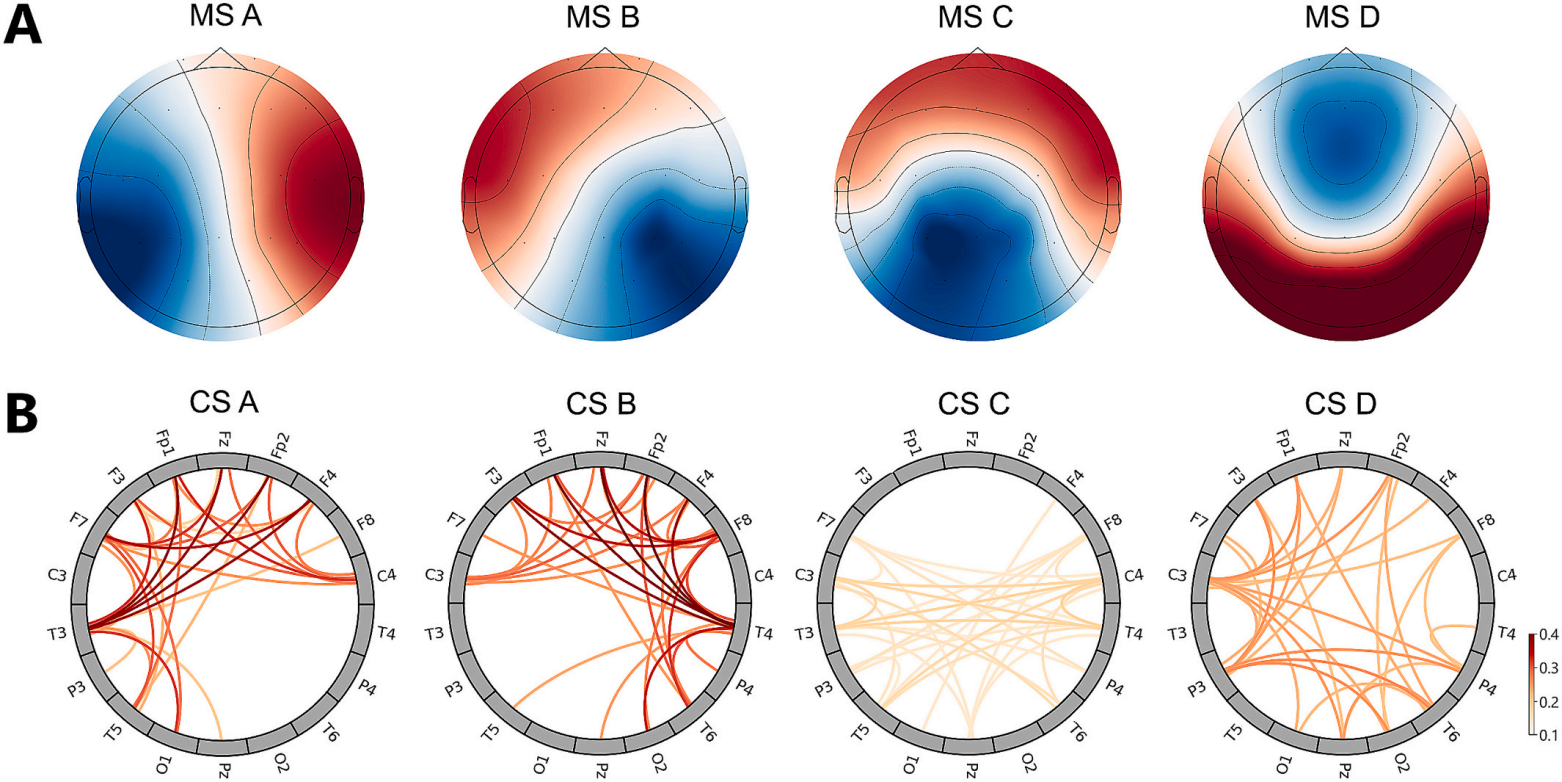
Global microstates (MS) and connectivity states (CS). A. Global microstates computed across all subjects and resting-state sessions. B. Global connectivity states computed across all subjects and resting-state sessions. The edge of the circle is divided into electrodes with frontal electrodes placed at the top and occipital electrodes at the bottom. Lines inside the circle represent connectivity between to electrodes measured by ciPLV. Only the strongest 30% connections were plotted. Colors indicate connectivity strength.

The global microstates and connectivity states computed over all subjects and sessions are illustrated in Fig. 2. Microstates showed high topographical similarity to the four canonical microstates reported in the literature (Koenig et al., 2002; Musaeus et al., 2019).

The connectivity states represent connectivity between electrodes measured by ciPLV (Fig. 2B), where 0 indicates complete asynchrony of phases and 1 indicates perfect synchrony. Relatively high and focal connectivity can be seen between bifrontal and left-temporal electrodes in connectivity state A (ciPLV range 0.25 to 0.35) and between bifrontal and right-temporal electrodes in connectivity state B (ciPLV range 0.25 to 0.41). Connectivity state C (ciPLV range 0.11 to 0.13) shows relatively weak and diffuse connectivity spreading from the horizontal midline towards the occipital electrodes. Connectivity state D (ciPLV range 0.20 to 0.25) is characterized by medium strength, broadly distributed connectivity without a clear regional focus.

### 3.2 Group and session effects

Table 2 summarizes the main effects of subject group and RS session, and their interaction effect, on microstate and connectivity state variables. The RS sessions consisted of RS1 (before memory task), RS2-SHAM (after memory task and sham stimulation), and RS2-STIM (after memory task and rTMS). All significant main and interaction effects were followed by post-hoc analyses (detailed in Supplementary Table 1 and 2). In the main text, we prioritized the reporting of interaction effects. Main effects were only elaborated on if the interaction effect was non-significant. Supplementary Fig. 4 shows the main effects of group on microstates and connectivity states without accounting for interaction effects.

**Table 2:** Robust linear mixed-effects model (RLMM) results for the effects of subject group and resting-state sessions on microstate / connectivity state variables.

		Duration			Coverage		
		*df*	*Χ*^*2*^	*P-value*⁎	*df*	*Χ*^*2*^	*P-value*⁎
Microstates (MS)							
MS A	Group	1	0.769	0.507	1	5.320	0.070
	Session	2	1.120	0.685	2	15.218	**0.002**
	Group × Session	2	0.334	0.903	2	0.022	0.989
MS B	Group	1	1.651	0.312	1	0.044	0.903
	Session	2	5.578	0.124	2	2.886	0.354
	Group × Session	2	17.304	**0.001**	2	24.502	**<0.001**
MS C	Group	1	0.136	0.814	1	4.647	0.079
	Session	2	2.538	0.409	2	0.154	0.966
	Group × Session	2	5.196	0.143	2	4.452	0.199
MS D	Group	1	5.131	0.070	1	4.511	0.079
	Session	2	3.264	0.312	2	0.030	0.989
	Group × Session	2	5.744	0.123	2	7.240	0.076

Connectivity states (CS)							
CS A	Group	1	0.485	0.609	1	13.078	**0.002**
	Session	2	3.202	0.312	2	1.696	0.556
	Group × Session	2	8.264	0.056	2	17.102	**0.001**
CS B	Group	1	0.939	0.469	1	28.088	**<0.001**
	Session	2	6.738	0.079	2	31.074	**<0.001**
	Group × Session	2	21.556	**<0.001**	2	16.014	**0.002**
CS C	Group	1	2.478	0.205	1	5.776	0.060
	Session	2	4.126	0.218	2	0.752	0.804
	Group × Session	2	7.002	0.079	2	7.632	0.070
CS D	Group	1	0.777	0.507	1	40.142	**<0.001**
	Session	2	1.408	0.609	2	37.826	**<0.001**
	Group × Session	2	0.532	0.855	2	13.429	**0.005**

*Χ*^*2*^: Chi square, df: Degree of freedom.

⁎ *P*-values were corrected for multiple comparisons using the Benjamini-Hochberg FDR method (Benjamini and Hochberg, 1995). For significant effects, pairwise post-hoc comparisons were conducted using estimated marginal means, and can be found in Supplementary Table 1 (microstates) and Supplementary Table 2 (connectivity states).

#### 3.2.1 Group and session effects on microstates

**Fig. 3:**
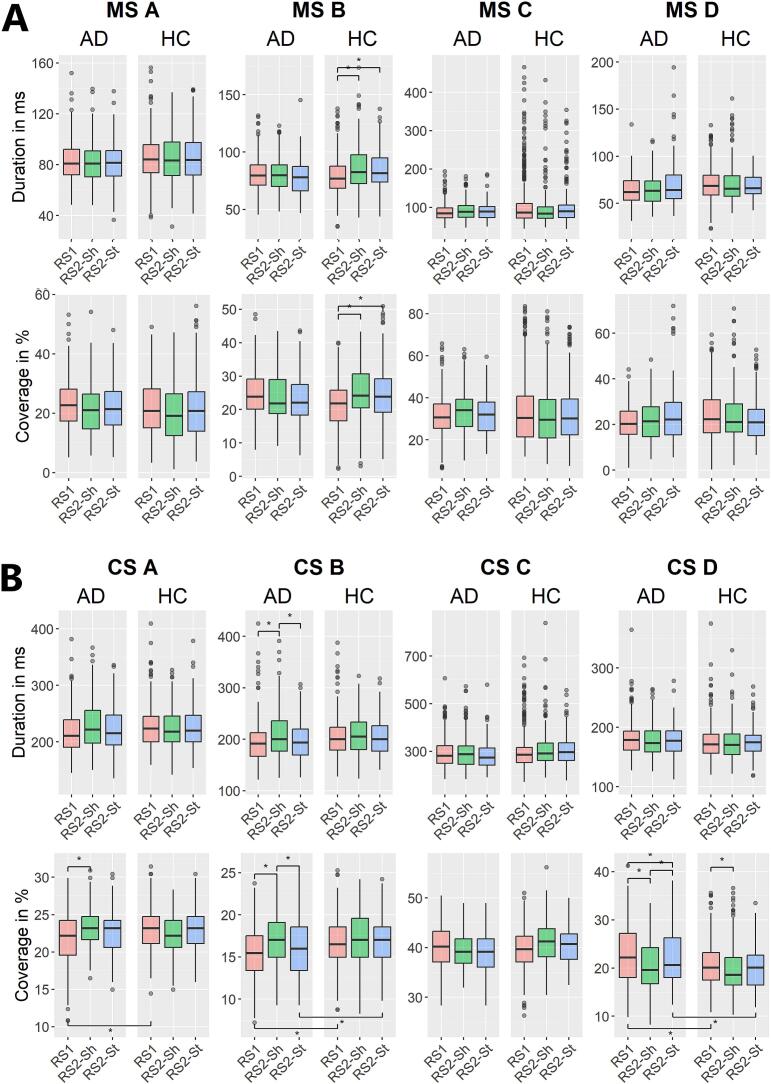
Group × resting-state (RS) session interaction effects on microstates (MS) and connectivity states (CS) Significant differences were marked with an asterisk (*). A. In the HC group, duration and coverage of microstate B were higher after the memory task with (RS2-STIM) or without rTMS (RS2-SHAM) compared to baseline (RS1). There were no between-session differences in the AD group. B. Coverage of connectivity states A, B and D differed between AD and HC during baseline (RS1). After the memory task without rTMS (RS2-SHAM), coverage of connectivity states A and B in the AD group increased and did not differ from the HC group anymore. Likewise, coverage of connectivity state D decreased in the AD group during RS2-SHAM and did not differ from the HC group anymore. With rTMS (RS2-STIM), this shift in the AD group did not occur.

For microstates, the group × session interaction affected the duration and coverage of microstate B (Fig. 3A). This interaction was driven by an increase in both microstate B variables from RS1 to RS2 (with and without rTMS) in the HC group, which was absent in the AD group: In the HC group, the duration of microstate B was lower in RS1 compared to RS2-SHAM (*p* < 0.001, ꞵ = −0.077, CI [−0.12, −0.03], ES = −0.351) and RS2-STIM (*p* = 0.002, ꞵ = −0.066, CI [−0.11, −0.02], ES = −0.302). The coverage of microstate B was also lower in RS1 compared to RS2-SHAM (p < 0.001, ꞵ = −0.148, CI [−0.23, −0.06], ES = −0.370) and RS2-STIM (*p* = 0.001, ꞵ = −0.123, CI [−0.20, −0.04], ES = −0.307) in the HC group. There were no session-related effects in the AD group. Aside from this interaction, microstate A coverage was affected by session regardless of subject group (Table 2). Post-hoc analysis (Supplementary Table 1) revealed that microstate A coverage was higher in RS1 than in RS2-SHAM (*p* < 0.001, ꞵ = 0.097, CI [0.04, 0.16], ES = 0.242).

#### 3.2.2 Group and session effects on connectivity states

The group × session interaction affected the coverage of connectivity states A, B, and D, and the duration of connectivity state B (Fig. 3B).

For connectivity state A coverage, a decomposition of the interaction revealed that RS1 was lower in the AD group than in the HC group (p < 0.001, ꞵ = −0.074, CI [−0.10, −0.05], ES = −0.457), and RS1 was lower than RS2-SHAM in the AD group (p = 0.001, ꞵ = −0.053, CI [−0.09, −0.02], ES = −0.329).

For connectivity state B, coverage was not only lower in the AD group compared to the HC group during RS1 (p < 0.001, ꞵ = −0.087, CI [−0.11, −0.06], ES = −0.541), but also during RS2-STIM (*p* = 0.003, ꞵ = −0.049, CI [−0.08, 0.02], ES = −0.302). In the AD group, coverage of connectivity state B was lower in RS1 than RS2-SHAM (p < 0.001, ꞵ = −0.099, CI [−0.13, −0.06], ES = −0.612) and higher in RS2-SHAM than RS2-STIM (p = 0.001, ꞵ = 0.062, CI [0.02, 0.10], ES = 0.384). This was also the case for the duration of connectivity state B (RS1 vs RS2-SHAM: p < 0.001, ꞵ = −0.074, CI [−0.11, −0.04], ES = −0.460; RS2-SHAM vs RS2-STIM: *p* = 0.026, ꞵ = 0.044, CI [0.00, 0.08], ES = 0.274).

As for connectivity state D, coverage was higher in the AD group compared to the HC group during RS1 (p < 0.001, ꞵ = 0.104, CI [0.08, 0.13], ES = 0.644) and RS2-STIM (p < 0.001, ꞵ = 0.079, CI [0.05, 0.11], ES = 0.487). In the AD group, coverage also differed between all three sessions, i.e., between RS1 and RS2-SHAM (p < 0.001, ꞵ = 0.109, CI [0.07, 0.14], ES = 0.674), between RS1 and RS2-STIM (*p* = 0.002, ꞵ = 0.050, CI [0.01, 0.09], ES = 0.310), and between RS2-SHAM and RS2-STIM (p = 0.002, ꞵ = −0.059, CI [−0.10, −0.02], ES = −0.364) with RS2-SHAM having the lowest coverage. Coverage of connectivity state D also showed the only between-session difference in the HC group, which was RS1 vs RS2-SHAM (p < 0.001, ꞵ = 0.061, CI [0.03, 0.09], ES = 0.377).

In summary, the interaction effects on connectivity states A, B, and D were primarily driven by two patterns: 1. Between-group differences during RS1 and RS2-STIM, and 2. between-session differences in the AD group that were largely absent in the HC group. Whereas the coverage of connectivity states A and B was lower in AD than HC at baseline (RS1), it increased in the AD group after the memory task without rTMS (RS2-SHAM) to an extent where it did not differ from the HC group anymore (Fig. 3B). With concurrent rTMS (RS2-STIM), however, this alignment effect was diminished in connectivity state A. For connectivity state B, this alignment effect did not occur, as the between-group difference disappeared in RS2-SHAM but remained in RS2-STIM. The same was observed for connectivity state D, which mirrored the pattern of connectivity state B in the opposite direction (i.e., reduced coverage only after the memory task without rTMS). rTMS thus appeared to prevent the task-induced alignment of connectivity states B and D in our AD patients.

### 3.3 Correlation with memory task performance

**Fig. 4:**
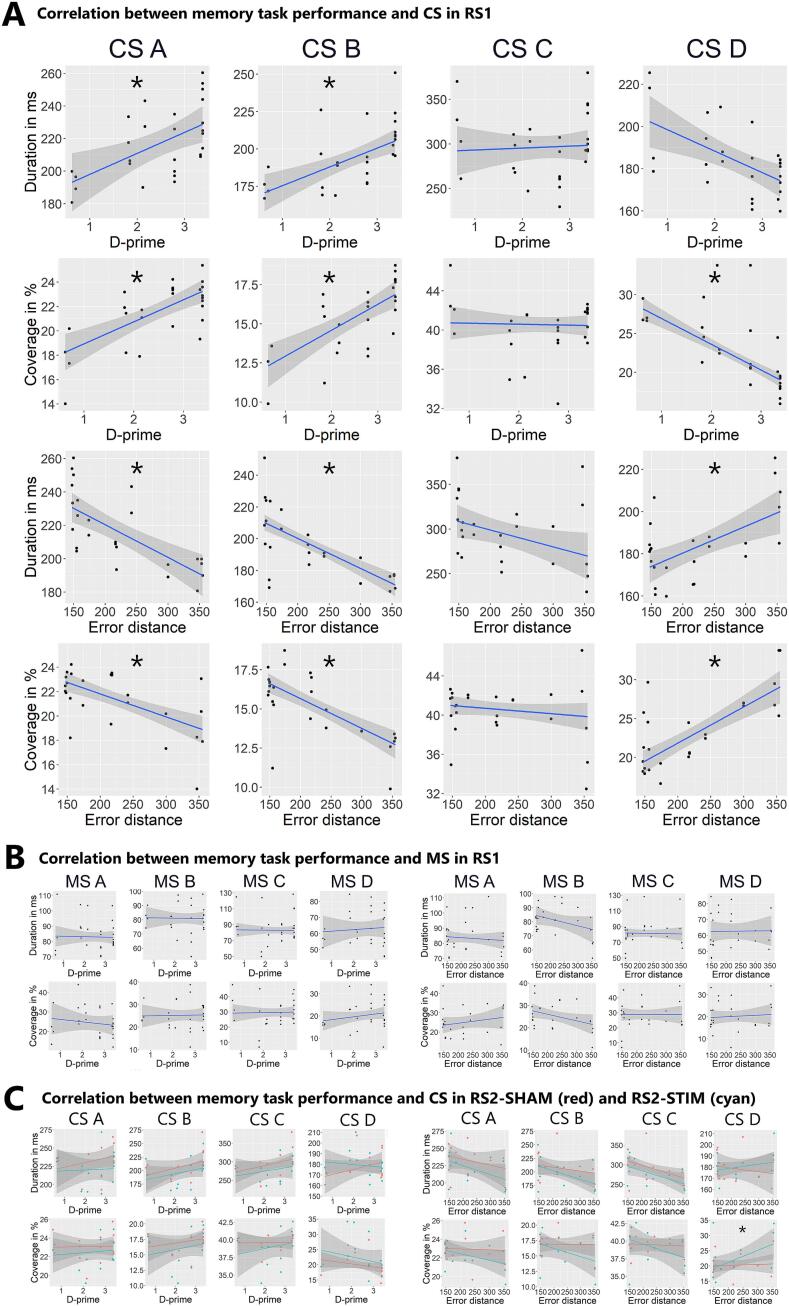
Effect of memory task performance on connectivity states (CS) and microstates (MS) High d-prime and low error distance indicate a good performance in the visuospatial memory task. Significant values were marked with an asterisk (*). A. During the baseline session (RS1), connectivity states A and B were positively correlated with task performance, while connectivity state D was negatively correlated with task performance. B. There was no correlation between microstates and task performance. C. After the memory task (RS2-SHAM and RS2-STIM), coverage of connectivity state D was negatively correlated with task performance.

The statistical results for RS1 were summarized in Table 3 and plotted in Fig. 4A and B. The statistical results for RS2 were detailed in Supplementary Table 3 and plotted in Fig. 4C. Note that higher d′ and lower error distance indicate better performance in the visuospatial memory task.

**Table 3:** Robust linear mixed-effects model (RLMM) results for the effects of memory task performance on microstate / connectivity state variables during RS1.

		Duration			Coverage		
		*df*	*Χ*^*2*^	*P-value*⁎	*df*	*Χ*^*2*^	*P-value*⁎
Microstates (MS)							
MS A	*d’*	1	0.013	0.910	1	1.985	0.299
	Error distance	1	1.667	0.331	1	0.029	0.910
MS B	*d’*	1	0.051	0.910	1	0.110	0.910
	Error distance	1	5.067	0.053	1	5.140	0.053
MS C	*d’*	1	0.042	0.910	1	0.013	0.910
	Error distance	1	0.738	0.568	1	0.063	0.910
MS D	*d’*	1	1.586	0.333	1	3.930	0.101
	Error distance	1	0.145	0.910	1	1.854	0.308

Connectivity states (CS)							
CS A	*d’*	1	6.305	**0.032**	1	19.287	**<0.001**
	Error distance	1	9.112	**0.010**	1	17.095	**<0.001**
CS B	*d’*	1	8.597	**0.012**	1	29.161	**<0.001**
	Error distance	1	9.946	**0.007**	1	34.247	**<0.001**
CS C	*d’*	1	0.470	0.686	1	0.065	0.910
	Error distance	1	5.207	0.053	1	0.824	0.555
CS D	*d’*	1	2.453	0.235	1	53.843	**<0.001**
	Error distance	1	6.632	**0.032**	1	78.954	**<0.001**

*Χ*^*2*^: Chi square, df: Degree of freedom.

⁎ P-values were corrected for multiple comparisons using the Benjamini-Hochberg FDR method (Benjamini and Hochberg, 1995).

Both performance scores were correlated with connectivity states A, B and D in the baseline session (RS1, Fig. 4A). Higher d′ and lower error distance were correlated with higher duration and coverage of connectivity states A and B. Connectivity state D was correlated with both performance scores in the opposite direction: Higher d′ was correlated with lower coverage of connectivity state D, while lower error distance was correlated with lower duration and coverage of connectivity state D. Notably, connectivity states A, B, and D represent the connectivity states where the coverage differs between the AD and HC group during the baseline session. Taken together, these correlations suggest that AD patients whose baseline connectivity state variables more closely resembled those of the HC group also performed better in the memory task. No effects of memory task performance on microstates were found (Fig. 4B).

After the memory task, only the coverage of connectivity state D showed a positive correlation with error distance (*Χ*^*2*^(1): 10.738, *p* = 0.034) (Fig. 4C). As the regression slopes in Fig. 4C for connectivity state D coverage suggested a stronger correlation to error distance during RS2-STIM (cyan) compared to RS2-SHAM (red), we further evaluated the effect of the session × error distance interaction on connectivity state D coverage. This interaction was not significant after correcting for multiple comparisons (*Χ*^*2*^(1): 5.831, *p* = 0.284). Other than that, there were no correlations between connectivity states and task performance in RS2-STIM and RS2-SHAM.

### 3.4 Correlation with amyloid status

**Fig. 5:**
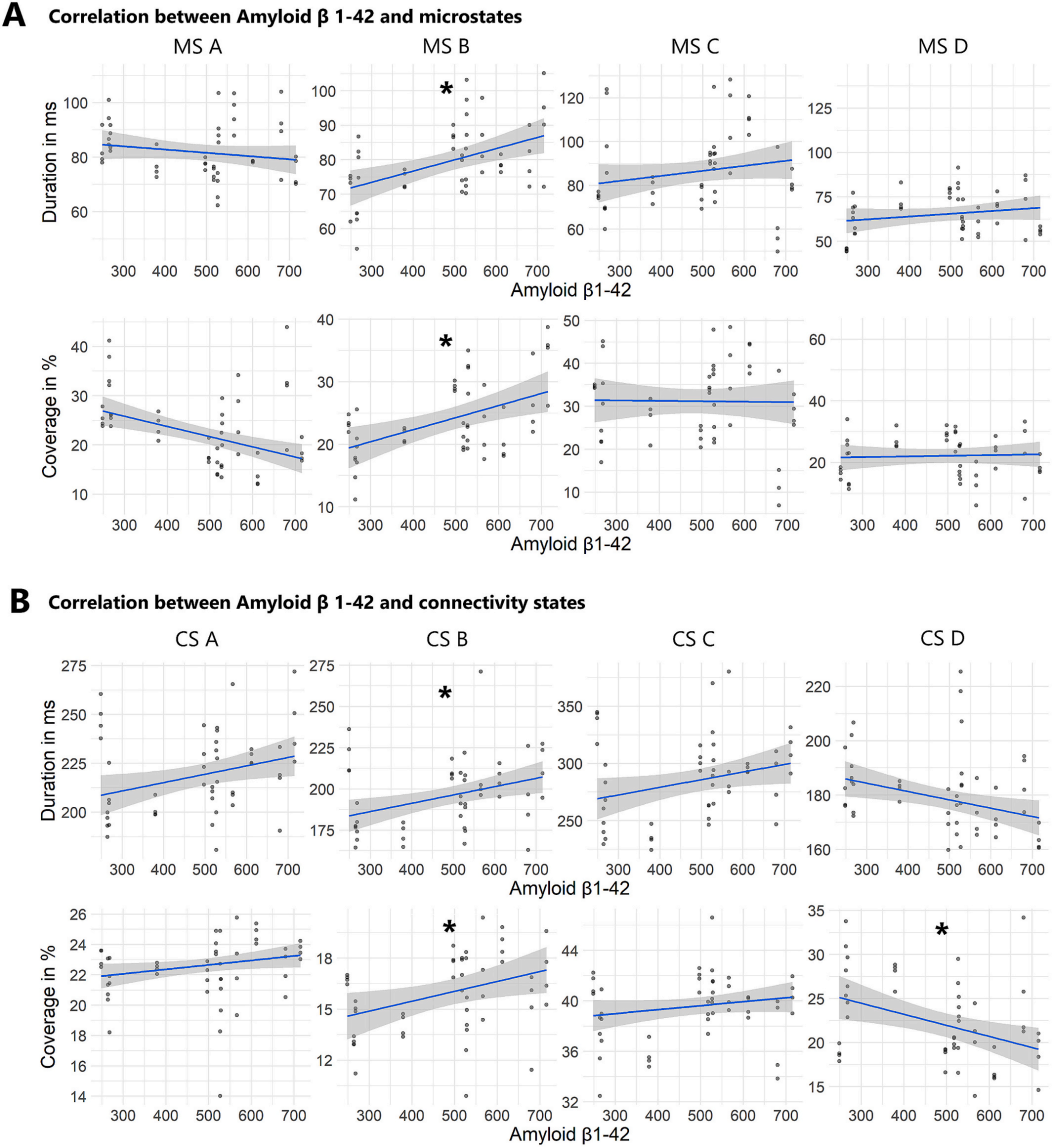
Correlation between Aβ_42_ and microstate and connectivity state variables. Significant values were marked with an asterisk (*). **A.** Duration and coverage of microstate B were positively correlated with Aβ_42_. **B**. Duration and coverage of connectivity state B were positively correlated with Aβ_42_, while coverage of connectivity state D was negatively correlated with Aβ_42_.

Supplementary Table 4 contains the CSF Aβ_42_ levels available for 12 out of 15 patients in the AD group, and the time interval between CSF collection and RS-EEG recording. These intervals ranged from <1 month to 6.3 years, with a median of 9.5 months. The statistical results regarding the effect of Aβ_42_ on microstates and connectivity states during RS1 were summarized in Table 4 and plotted in Fig. 5.

**Table 4:** Robust linear mixed-effects model (RLMM) results for the effects of Aβ_42_ level in cerebrospinal fluid on microstate / connectivity state variables.

		Duration			Coverage		
		*df*	*Χ*^*2*^	*P-value*⁎	*df*	*Χ*^*2*^	*P-value*⁎
Microstates (MS)							
MS A	Aβ_42_	1	0.313	0.658	1	5.223	0.056
MS B	Aβ_42_	1	5.810	**0.043**	1	7.185	**0.029**
MS C	Aβ_42_	1	2.954	0.152	1	0.000	0.989
MS D	Aβ_42_	1	2.425	0.191	1	0.215	0.686

Connectivity states (CS)							
CS A	Aβ_42_	1	3.924	0.095	1	1.412	0.313
CS B	Aβ_42_	1	6.388	**0.037**	1	10.296	**0.010**
CS C	Aβ_42_	1	4.059	0.095	1	0.666	0.510
CS D	Aβ_42_	1	1.559	0.308	1	26.242	**<0.001**

*Χ*^*2*^: Chi square, df: Degree of freedom.

⁎ P-values were corrected for multiple comparisons using the Benjamini-Hochberg FDR method (Benjamini and Hochberg, 1995).

The duration and coverage of microstate B were positively correlated with Aβ_42_ (Fig. 5A). Similarly, the duration and coverage of connectivity state B showed a positive correlation with Aβ_42_ (Fig. 5B). For connectivity state D, there was a negative correlation between coverage and Aβ_42_ (Fig. 5B). Notably, while the correlation between connectivity states B and D with Aβ_42_ followed the same pattern as their correlation to memory task performance (Fig. 4A), there was no direct relationship between Aβ_42_ and memory task performance: Pearson's correlation indicated no significant correlation between Aβ_42_ and d′ (r = −0.067, CI [−0.35, 0.23], *p* = 0.663), nor between Aβ_42_ and error distance (r= −0.187, CI [−0.47, 0.13], *p* = 0.247). These results imply that connectivity states may act as a more sensible marker for amyloid-related cognitive changes than CSF amyloid levels alone. However, these findings should be interpreted with caution, given the limited availability of Aβ_42_ parameters (*n* = 12) and the large variability in the time interval between CSF collection and RS-EEG recording in our study.

## 4 Discussion

In this study, we analyzed microstates and connectivity states derived from RS-EEG in AD patients and healthy controls (HC). Prior studies comparing microstates in AD patients and healthy cohorts have reported both, differences in the topographical configuration of some microstates, as well as differences in temporal dynamics (Dierks et al., 1997; Tait et al., 2020). The choice to employ cross-group or group-specific microstates and connectivity states should be determined by both data-driven validation and the requirements of the research question. In our subjects, group-specific microstates and connectivity states demonstrated high topographical similarity between the AD and HC groups (Supplementary Fig. 2 and 3). Our AD patients were clinically in the stages of MCI to mild dementia. As microstates and connectivity states reflect “poles” of brain activity, it is plausible that AD in its early stages does not fundamentally reshape these states, but rather manifests as disturbances in their temporal sequence. Consequently, we computed one set of microstates and connectivity states across all sessions and groups (Fig. 2), which enabled a direct comparison of the temporal dynamics between the AD and HC groups. The sessions included recordings before and after a visuospatial memory task, both with and without concurrent rTMS. Microstates showed task-related differences in the HC group (Fig. 3A), while connectivity states predominately showed task-related differences in the AD group (Fig. 3B). Only connectivity states displayed group differences, which were observable during the baseline session and after the memory task with rTMS (Fig. 3B). Furthermore, connectivity states, but not microstates, were correlated with memory task performance in the AD group (Fig. 4).

One of the objectives of this study was to compare our RS-EEG results to findings from RS-fMRI studies. Our study unveiled lower coverages of connectivity states A and B, which represent high and focal connectivity, and higher coverage of connectivity state D, which depicts medium and diffuse connectivity, in AD compared to HC, during the baseline session (RS1). Furthermore, while group-specific connectivity states were largely consistent, connectivity state D exhibited a subtle left-hemispheric accentuation in the HC group that appeared more diffuse in the AD group (Supplementary Fig. 3). These results align with fMRI studies suggesting a shift towards lower, more diffuse connectivity in patients with AD and subjective cognitive decline (SCD) (Chen et al., 2021; Schumacher et al., 2019a). The fMRI studies also reported that connectivity states were more sensitive than static graph measures in detecting differences between AD/SCD patients and healthy controls. In Chen et al., the mean duration of the connectivity state representing focal, high connectivity was positively correlated with memory performance in their SCD group, a finding we also observed in our AD patients. Nevertheless, other fMRI studies reported contrasting results with decreased connectivity states representing hypoconnectivity (Gu et al., 2020; Zang et al., 2024). It should be noted that cross-modality comparisons of connectivity states are complicated by inconsistencies in connectivity measures and variance-dependent state numbers. Further fMRI and EEG studies are thus warranted to compare connectivity states across modalities and computational methods in AD.

Our data confirmed the hypothesis on higher task-related sensitivity of connectivity states in the AD group. Connectivity states A, B and D unveiled task-related differences in the AD group (Fig. 3B), with values shifting towards those observed in the HC group after the memory task without rTMS. In contrast, the HC group only showed task-related alterations in the coverage of connectivity state D. This finding supports prior evidence that less task-related alterations in functional connectivity correspond to better cognitive performance (Cassady et al., 2023; Schultz and Cole, 2016). One of the studies even described an association between higher levels of tau in regions of the episodic memory network with increased task-related alterations in functional connectivity in healthy individuals (Cassady et al., 2023), suggesting a direct link between AD pathology and decreased memory network stability. Both studies related their results to the “neural efficiency hypothesis” (Neubauer and Fink, 2009), which states that more efficient brain network optimization during tasks is linked to better performance. In our study, both task performance scores were correlated with several connectivity states at baseline, while correlations were largely absent after the memory task (Fig. 4B). This task-related flexibility in the connectivity states of our AD patients thus seems to represent a reactive phenomenon to the memory task that did not yield a direct behavioral advantage, as the task performance in the patients primarily depended on how closely their baseline connectivity states resembled the efficiently optimized network of the healthy population. These results suggest that the post-task alignment of connectivity states can be seen as a marker of neural (in-)efficiency rather than a compensatory mechanism.

Microstates, on the other hand, mirrored findings from prior research in healthy subjects. Notably, there was a large variance in the duration of microstate C in the HC group (Fig. 3A). This has been described before and attributed to the intra- and inter-subject variability of alpha power that modulates the duration of microstate C (Croce et al., 2020). Furthermore, microstate B variables were higher after the (visuospatial) memory task, reflecting its association with the visual RSN and visual imagery (Britz et al., 2010; Michel and Koenig, 2018).

Regarding rTMS, our hypothesis was not confirmed: Neither microstate B, which is associated with the visual RSN, nor microstate D, which topographically represents increased posterior EEG power, showed an increase induced by rTMS that was distinguishable from the effect of the memory task alone (Fig. 3A). Moreover, rTMS seemed to interfere with task-related alterations in connectivity states: While connectivity states in the AD group aligned with values comparable to the HC group after the memory task alone, this shift was reduced or absent with concurrent rTMS (Fig. 3B). This finding can be interpreted in two ways: According to the neural efficiency hypothesis, reduced task-related alterations may reflect increased network stability. However, if the post-task alignment represents a “necessary” responsive phenomenon to task engagement, its absence suggests failed adaptation. But most importantly, a previous study conducted on our dataset observed that rTMS did not affect the memory task performance (Fassbender et al., 2026). We therefore conclude that the task- and rTMS-related alterations in connectivity states in the AD group reflect short-term changes in network configurations without direct behavioral effects.

### 4.1 Limitations

A limitation of our study is the relatively small sample size (*N* = 15). However, unlike the majority of studies examining microstates in patients with AD, which typically employ between-subject comparisons of single-session EEG recordings (Lassi et al., 2023; Lian et al., 2021; Tait et al., 2020), our study utilized a 2 × 2 repeated-measures design. This within-subject design controls for individual baseline differences and thereby enhances statistical power. We also utilized RLMMs with multiple observations per subject. This approach increases the effective sample size and statistical power, with the added benefit of integrating intra-subject variance into the model. At the same time, this robust estimation method reduces the influence of outliers, thereby balancing the risk of Type I and Type II errors. Nevertheless, the sample size must be taken into consideration regarding the generalizability of our data.

Another limitation of our study lies in the application of a threshold to the functional connectivity matrices when computing connectivity states. While thresholding is a common practice to reduce the influence of spurious, weak connections (Rubinov and Sporns, 2010), the selection of the specific threshold is dataset-dependent. In our study, we applied a threshold of 30% to achieve an optimal balance between noise reduction and the preservation of non-fragmented connectivity clusters. The Calinski-Harabasz score (Calinski and Harabasz, 1974) further confirmed high between-cluster separation and within-cluster consistency at this specific threshold. Ultimately, the lack of a standardized thresholding method must be considered as a potential confound when comparing functional connectivity states across studies.

## 5 Conclusion

Phase-based connectivity states captured disease-related differences between AD patients and healthy controls. They were also more sensitive than amplitude-based microstates to short-term, task-related network changes in AD patients, which may be due to the decrease of alpha amplitude in relation to disease progression in AD. Overall, connectivity states indicated a shift towards lower and more diffuse connectivity and decreased network stability in AD patients, which correlated with their memory performance. EEG-derived connectivity states thus provide a powerful, cost-efficient tool for assessing network alterations and cognitive functions in AD, demonstrating their potential as a sensitive biomarker of disease progression.

## Author Contribution

JWK performed the analytical calculations and drafted the manuscript. AY and NR developed and verified the analytical methods. CK, RVF and OAO contributed to the planning and data collection of the study that generated the data used in the present analyses. OAO and SD devised the main conceptual ideas. SD and GRF supervised the project. All authors discussed the results and contributed to the final manuscript.

## Declaration of competing interest

The authors declare that they have no known competing financial interests or personal relationships that could have appeared to influence the work reported in this paper.

## Data Availability

The data that support the findings of this study are not openly available, as the participants of the study did not give written consent for their data to be shared publicly.
