# Supplementary material for: Microstates and connectivity states reveal task-related network reorganization in Alzheimer's disease patients
Source: Clin Neurophysiol Pract. 2026 Jul 28;11:664–76. doi: 10.1016/j.cnp.2026.07.016 (PMC13473952; doi:10.1016/j.cnp.2026.07.016)
Supplement: Supplementary file 1 [file mmc1.docx]

**Supplementary Data for**

**Microstates and connectivity states reveal task-related network reorganization
in Alzheimer’s disease patients**

Ji-Won Kim ^a,b^, Azamat Yeldesbay ^a,c^, Christina Kehm ^b^, Ronja V. Fassbender ^b^, Oezguer A. Onur ^b^, Nils Rosjat ^a^, Gereon R. Fink ^a,b^, Silvia Daun ^a,c^

^a^ Cognitive Neuroscience, Institute of Neuroscience & Medicine (INM-3), Forschungszentrum Jülich

^b^ Department of Neurology, Faculty of Medicine, University Hospital Cologne

^c^ Institute of Zoology, University of Cologne

**Supplementary Figure 1. Topographical similarity of microstates across studies**

Once four global microstates were obtained, we compared their topographies to those from selected published studies using the MATLAB-based application MS-Template-Explorer (Koenig et al., 2024). This was done to validate the visual labeling of our microstates and enhance the objectivity of the labeling process. Lassi et al. (2023) and Musaeus et al. (2019) compared patients within the Alzheimer’s disease continuum with healthy controls, similar to our study. Koenig et al. (2002) was included as a reference for microstate topographies in healthy individuals. The numbers in the colored fields represent the percentage of topographical similarity, or shared variance, between microstates from our study and microstates from the studies labeled along the x-axis. Black fields indicate similarity values below 50%. In all three comparisons, the highest similarity values lie along the diagonal axis, indicating similar topographies and order of microstates.

**Supplementary Figure 2. Topographical similarity of microstates in AD and HC**

**A/B:** Microstates were computed separately for the HC group and the AD group for topographical comparison. As the clustering algorithm disregards polarity, the orientation of the electric field (red vs blue) is arbitrary.

**C:** The numbers in the colored fields represent the percentage of topographical similarity, or shared variance, between microstates from the HC group and the AD group, as computed by MS-Template-Explorer (Koenig et al., 2024). Black fields indicate similarity values below 50%. The comparison revealed very high topographical similarities between the two groups in all four microstate pairs.

**Supplementary Figure 3. Topographical similarity of connectivity states in AD and HC**

**A/B:** Connectivity states were computed separately for the HC group and the AD group for topographical comparison. Only the strongest 30% connections were plotted.

**C:** The numbers represent Pearson’s correlations coefficients between connectivity states from the HC group and the AD group. Pairs of matching connectivity states were marked yellow. The comparison revealed strong correlations between the two groups in all four connectivity state pairs.

**Supplementary Figure 4. Group comparison of microstates (MS) and connectivity states (CS)**

Significant differences were marked with an asterisk (*).

**A**. There were no differences in the duration and coverage of microstates between groups.

**B**. The coverage of connectivity states A, B and D was higher in AD than HC across sessions. There were no group differences in the duration of connectivity states. Note: These comparisons do not account for group × session interactions (see Fig. 4 for interaction effects).

**Supplementary Table 1. Results from pairwise post-hoc analyses for microstates (MS) duration and coverage**

|  | | | **Duration** | | | | **Coverage** | | | |
| --- | --- | --- | --- | --- | --- | --- | --- | --- | --- | --- |
|  |  |  | *p* | *ꞵ* | *CI* | *ES* | *p* | *ꞵ* | *CI* | *ES* |
| **MS A** | AD vs HC | | n.a. | n.a. | n.a. | n.a. | n.a. | n.a. | n.a. | n.a. |
|  | RS1 vs RS2-SHAM | | n.a. | n.a. | n.a. | n.a. | **<0.001** | 0.097 | [0.04, 0.16] | 0.242 |
|  | RS1 vs RS2-STIM | | n.a. | n.a. | n.a. | n.a. | 0.079 | 0.056 | [-0.00, 0.12] | 0.142 |
|  | RS2-SHAM vs RS2-STIM | | n.a. | n.a. | n.a. | n.a. | 0.510 | -0.040 | [-0.11, 0.03] | -0.101 |
|  | All interactions | | n.a. | n.a. | n.a. | n.a. | n.a. | n.a. | n.a. | n.a. |
| **MS B** | AD vs HC | | n.a. | n.a. | n.a. | n.a. | n.a. | n.a. | n.a. | n.a. |
|  | RS1 vs RS2-SHAM | | n.a. | n.a. | n.a. | n.a. | n.a. | n.a. | n.a. | n.a. |
|  | RS1 vs RS2-STIM | | n.a. | n.a. | n.a. | n.a. | n.a. | n.a. | n.a. | n.a. |
|  | RS2-SHAM vs RS2-STIM | | n.a. | n.a. | n.a. | n.a. | n.a. | n.a. | n.a. | n.a. |
|  | **RS1** AD vs HC | | 0.483 | 0.023 | [-0.04, 0.09] | 0.106 | 0.071 | 0.081 | [ 0.00, 0.16] | 0.142 |
|  | **RS2-SHAM** AD vs HC | | 0.079 | -0.065 | [-0.14, 0.01] | -0.295 | 0.076 | -0.077 | [-0.16, 0.01] | -0.191 |
|  | **RS2-STIM** AD vs HC | | 0.061 | -0.079 | [-0.15, 0.01] | -0.360 | 0.091 | -0.072 | [-0.16, 0.01] | -0.180 |
|  | **AD** | RS1 vs RS2-SHAM | 0.853 | 0.011 | [-0.04, 0.06] | 0.050 | 0.222 | 0.062 | [-0.03, 0.15] | 0.154 |
|  |  | RS1 vs RS2-STIM | 0.185 | 0.036 | [-0.01, 0.08] | 0.163 | 0.069 | 0.082 | [ 0.00, 0.17] | 0.206 |
|  |  | RS2-SHAM vs RS2-STIM | 0.535 | 0.025 | [-0.03, 0.08] | 0.113 | 0.878 | 0.021 | [-0.08, 0.12] | 0.052 |
|  | **HC** | RS1 vs RS2-SHAM | **<0.001** | -0.077 | [-0.12, -0.03] | -0.351 | **<0.001** | -0.148 | [-0.23, -0.06] | -0.370 |
|  |  | RS1 vs RS2-STIM | **0.002** | -0.066 | [-0.11, -0.02] | -0.302 | **0.001** | -0.123 | [-0.20, -0.04] | -0.307 |
|  |  | RS2-SHAM vs RS2-STIM | 0.879 | 0.011 | [-0.04, 0.06] | 0.049 | 0.811 | 0.025 | [-0.07, 0.12] | 0.063 |
| **MS C** | All pairwise comparisons | | n.a. | n.a. | n.a. | n.a. | n.a. | n.a. | n.a. | n.a. |
| **MS D** | All pairwise comparisons | | n.a. | n.a. | n.a. | n.a. | n.a. | n.a. | n.a. | n.a. |

Post-hoc pairwise comparison of main and interaction effects from the robust linear mixed-effects models. In cases where the initial Wald Chi-square test for a main or interaction effect was not significant, post-hoc analysis was not applied (“n.a.”). P-values were adjusted for multiple comparisons using the Tukey method.

AD: Alzheimer’s disease, CI: Confidence interval, CS: Connectivity states, ES: Effect size (Cohen’s d), HC: Healthy controls, MS: Microstates

**Supplementary Table 2**. **Results from pairwise post-hoc analyses for connectivity state (CS) duration and coverage**

|  | | | **Duration** | | | | **Coverage** | | | |
| --- | --- | --- | --- | --- | --- | --- | --- | --- | --- | --- |
|  |  |  | *p* | *ꞵ* | *CI* | *ES* | *p* | *ꞵ* | *CI* | *ES* |
| **CS A** | AD vs HC | | n.a. | n.a. | n.a. | n.a. | **<0.001** | -0.032 | [-0.05, -0.01] | -0.199 |
|  | RS1 vs RS2-SHAM | | n.a. | n.a. | n.a. | n.a. | n.a. | n.a. | n.a. | n.a. |
|  | RS1 vs RS2-STIM | | n.a. | n.a. | n.a. | n.a. | n.a. | n.a. | n.a. | n.a. |
|  | RS2-SHAM vs RS2-STIM | | n.a. | n.a. | n.a. | n.a. | n.a. | n.a. | n.a. | n.a. |
|  | **RS1** AD vs HC | | n.a. | n.a. | n.a. | n.a. | **<0.001** | -0.074 | [-0.10, -0.05] | -0.457 |
|  | **RS2-SHAM** AD vs HC | | n.a. | n.a. | n.a. | n.a. | 0.516 | 0.011 | [-0.02, 0.04] | 0.068 |
|  | **RS2-STIM** AD vs HC | | n.a. | n.a. | n.a. | n.a. | 0.054 | -0.037 | [-0.07, 0.00] | -0.208 |
|  | **AD** | RS1 vs RS2-SHAM | n.a. | n.a. | n.a. | n.a. | **0.001** | -0.053 | [-0.09, -0.02] | -0.329 |
|  |  | RS1 vs RS2-STIM | n.a. | n.a. | n.a. | n.a. | 0.091 | -0.031 | [-0.07, 0.00] | -0.195 |
|  |  | RS2-SHAM vs RS2-STIM | n.a. | n.a. | n.a. | n.a. | 0.415 | 0.022 | [-0.02, 0.06] | 0.134 |
|  | **HC** | RS1 vs RS2-SHAM | n.a. | n.a. | n.a. | n.a. | 0.069 | 0.032 | [ 0.00, 0.07] | 0.197 |
|  |  | RS1 vs RS2-STIM | n.a. | n.a. | n.a. | n.a. | 0.803 | 0.009 | [-0.02, 0.04] | 0.055 |
|  |  | RS2-SHAM vs RS2-STIM | n.a. | n.a. | n.a. | n.a. | 0.345 | -0.023 | [-0.06, 0.02] | -0.142 |
| **CS B** | AD vs HC | | n.a. | n.a. | n.a. | n.a. | **<0.001** | -0.047 | [-0.06, -0.03] | -0.292 |
|  | RS1 vs RS2-SHAM | | n.a. | n.a. | n.a. | n.a. | **<0.001** | -0.057 | [-0.08, -0.03] | -0.358 |
|  | RS1 vs RS2-STIM | | n.a. | n.a. | n.a. | n.a. | 0.262 | -0.017 | [-0.04, 0.01] | -0.109 |
|  | RS2-SHAM vs RS2-STIM | | n.a. | n.a. | n.a. | n.a. | **0.002** | 0.040 | [0.01, 0.07] | 0.249 |
|  | **RS1** AD vs HC | | 0.062 | -0.055 | [-0.11, 0.00] | -0.341 | **<0.001** | -0.087 | [-0.11, -0.06] | -0.541 |
|  | **RS2-SHAM** AD vs HC | | 0.924 | -0.003 | [-0.06, 0.06] | -0.019 | 0.759 | -0.005 | [-0.04, 0.03] | -0.032 |
|  | **RS2-STIM** AD vs HC | | 0.444 | -0.024 | [-0.09, 0.04] | -0.150 | **0.003** | -0.049 | [-0.08, -0.02] | -0.302 |
|  | **AD** | RS1 vs RS2-SHAM | **<0.001** | -0.074 | [-0.11, -0.04] | -0.460 | **<0.001** | -0.099 | [-0.13, -0.06] | -0.612 |
|  |  | RS1 vs RS2-STIM | 0.115 | -0.030 | [-0.06, 0.01] | -0.185 | 0.057 | -0.035 | [-0.07, 0.00] | -0.229 |
|  |  | RS2-SHAM vs RS2-STIM | **0.026** | 0.044 | [ 0.00, 0.08] | 0.274 | **0.001** | 0.062 | [ 0.02, 0.10] | 0.384 |
|  | **HC** | RS1 vs RS2-SHAM | 0.269 | -0.022 | [-0.06, 0.01] | -0.138 | 0.474 | -0.017 | [-0.05, 0.02] | -0.104 |
|  |  | RS1 vs RS2-STIM | 0.998 | 0.001 | [-0.03, 0.03] | 0.006 | 0.992 | 0.002 | [-0.03, 0.03] | 0.011 |
|  |  | RS2-SHAM vs RS2-STIM | 0.337 | 0.023 | [-0.02, 0.06] | 0.143 | 0.502 | 0.018 | [-0.02, 0.06] | 0.114 |
| **CS C** | All pairwise comparisons | | n.a. | n.a. | n.a. | n.a. | n.a. | n.a. | n.a. | n.a. |
| **CS D** | AD vs HC | | n.a. | n.a. | n.a. | n.a. | **<0.001** | 0.080 | [0.06, 0.10] | 0.493 |
|  | RS1 vs RS2-SHAM | | n.a. | n.a. | n.a. | n.a. | **<0.001** | 0.084 | [0.06, 0.11] | 0.526 |
|  | RS1 vs RS2-STIM | | n.a. | n.a. | n.a. | n.a. | **<0.001** | 0.037 | [0.01, 0.06] | 0.232 |
|  | RS2-SHAM vs RS2-STIM | | n.a. | n.a. | n.a. | n.a. | **<0.001** | -0.047 | [-0.07, -0.02] | -0.294 |
|  | **RS1** AD vs HC | | n.a. | n.a. | n.a. | n.a. | **<0.001** | 0.104 | [ 0.08, 0.13] | 0.644 |
|  | **RS2-SHAM** AD vs HC | | n.a. | n.a. | n.a. | n.a. | 0.096 | 0.030 | [0.00, 0.06] | 0.184 |
|  | **RS2-STIM** AD vs HC | | n.a. | n.a. | n.a. | n.a. | **<0.001** | 0.079 | [ 0.05, 0.11] | 0.487 |
|  | **AD** | RS1 vs RS2-SHAM | n.a. | n.a. | n.a. | n.a. | **<0.001** | 0.109 | [ 0.07, 0.14] | 0.674 |
|  |  | RS1 vs RS2-STIM | n.a. | n.a. | n.a. | n.a. | **0.002** | 0.050 | [ 0.01, 0.09] | 0.310 |
|  |  | RS2-SHAM vs RS2-STIM | n.a. | n.a. | n.a. | n.a. | **0.002** | -0.059 | [-0.10, -0.02] | -0.364 |
|  | **HC** | RS1 vs RS2-SHAM | n.a. | n.a. | n.a. | n.a. | **<0.001** | 0.061 | [ 0.03, 0.09] | 0.377 |
|  |  | RS1 vs RS2-STIM | n.a. | n.a. | n.a. | n.a. | 0.183 | 0.025 | [-0.01, 0.06] | 0.153 |
|  |  | RS2-SHAM vs RS2-STIM | n.a. | n.a. | n.a. | n.a. | 0.071 | -0.036 | [-0.07, 0.00] | -0.224 |

Post-hoc pairwise comparison of main and interaction effects from the robust linear mixed-effects models. In cases where the initial Wald Chi-square test for a main or interaction effect was not significant, post-hoc analysis was not applied (“n.a.”). P-values were adjusted for multiple comparisons using the Tukey method.

AD: Alzheimer’s disease, CI: Confidence interval, CS: Connectivity states, ES: Effect size (Cohen’s d), HC: Healthy controls, MS: Microstates

**Supplementary Table 3.** **Robust linear mixed-effects model (RLMM) results for the effects of memory task performance on microstate / connectivity state variables during RS2-SHAM and RS2-STIM**

|  | | **Duration** | | | **Coverage** | | |
| --- | --- | --- | --- | --- | --- | --- | --- |
|  |  | *df* | *Χ^2^* | *P-value** | *df* | *Χ^2^* | *P-value** |
| **Microstates (MS)** | | | | | | | |
| **MS A** | *d’* | 1 | 1.115 | 0.582 | 1 | 0.088 | 0.899 |
|  | Error distance | 1 | 0.709 | 0.711 | 1 | 0.129 | 0.899 |
| **MS B** | *d’* | 1 | 0.073 | 0.899 | 1 | 0.123 | 0.899 |
|  | Error distance | 1 | 2.902 | 0.352 | 1 | 3.699 | 0.349 |
| **MS C** | *d’* | 1 | 0.012 | 0.957 | 1 | 0.001 | 0.973 |
|  | Error distance | 1 | 2.607 | 0.352 | 1 | 1.792 | 0.413 |
| **MS D** | *d’* | 1 | 0.497 | 0.733 | 1 | 0.257 | 0.891 |
|  | Error distance | 1 | 2.554 | 0.352 | 1 | 3.685 | 0.349 |
| **Connectivity states (CS)** | | | | | | | |
| **CS A** | *d’* | 1 | 0.800 | 0.699 | 1 | 0.032 | 0.947 |
|  | Error distance | 1 | 1.809 | 0.413 | 1 | 0.580 | 0.714 |
| **CS B** | *d’* | 1 | 3.394 | 0.349 | 1 | 1.633 | 0.429 |
|  | Error distance | 1 | 4.278 | 0.349 | 1 | 2.716 | 0.352 |
| **CS C** | *d’* | 1 | 2.048 | 0.413 | 1 | 0.008 | 0.957 |
|  | Error distance | 1 | 1.795 | 0.413 | 1 | 0.113 | 0.899 |
| **CS D** | *d’* | 1 | 0.581 | 0.714 | 1 | 5.618 | 0.284 |
|  | Error distance | 1 | 0.074 | 0.899 | 1 | 10.738 | **0.034** |

*Χ^2^*: Chi square, df: Degree of freedom

* P-values were corrected for multiple comparisons using the Benjamini-Hochberg FDR method (Benjamini and Hochberg, 1995).

**Supplementary Table 4. Aβ42 level of each subject in the AD group* and time between cerebrospinal fluid (CSF) collection and RS-EEG**

| **Subject ID** | **Aβ42**  **(pg/ml)** | **Time between  CSF collection and RS-EEG (months)** |
| --- | --- | --- |
| **1** | 268.1 | 10 |
| **2** | 527 | 76 |
| **3** | 715.3 | 31 |
| **4** | 566.3 | 26 |
| **5** | 248.5 | 2 |
| **6** | 680.4 | 24 |
| **7** | 611.7 | 1 |
| **8** | 529 | 37 |
| **9** | 379.5 | 8 |
| **10** | 497.1 | 3 |
| **11** | 264 | 9 |
| **12** | 517.6 | 9 |
| **Median** | 522.3 | 9.5 |

***** Aβ42 values were available from 12 out of 15 patients in the AD group.

References

Benjamini Y, Hochberg Y. Controlling the False Discovery Rate: A Practical and Powerful Approach to Multiple Testing. Journal of the Royal Statistical Society Series B: Statistical Methodology 1995;57(1):289–300. https://doi.org10.1111/j.2517-6161.1995.tb02031.x.

Koenig T, Diezig S, Kalburgi SN, Antonova E, Artoni F, Brechet L, et al. EEG-Meta-Microstates: Towards a More Objective Use of Resting-State EEG Microstate Findings Across Studies. Brain topography 2024;37(2):218–31. https://doi.org10.1007/s10548-023-00993-6.

Koenig T, Prichep L, Lehmann D, Sosa PV, Braeker E, Kleinlogel H, et al. Millisecond by millisecond, year by year: normative EEG microstates and developmental stages. NeuroImage 2002;16(1):41–8. https://doi.org10.1006/nimg.2002.1070.

Lassi M, Fabbiani C, Mazzeo S, Burali R, Vergani AA, Giacomucci G, et al. Degradation of EEG microstates patterns in subjective cognitive decline and mild cognitive impairment: Early biomarkers along the Alzheimer's Disease continuum? NeuroImage. Clinical 2023;38:103407. https://doi.org10.1016/j.nicl.2023.103407.

Musaeus CS, Nielsen MS, Høgh P. Microstates as Disease and Progression Markers in Patients With Mild Cognitive Impairment. Frontiers in neuroscience 2019;13:563. https://doi.org10.3389/fnins.2019.00563.
